# A single point mutation in the *Plasmodium falciparum* 3′–5′ exonuclease does not alter piperaquine susceptibility

**DOI:** 10.1186/s12936-022-04148-z

**Published:** 2022-04-22

**Authors:** Nonlawat Boonyalai, Kirakarn Kirativanich, Chatchadaporn Thamnurak, Chantida Praditpol, Brian A. Vesely, Mariusz Wojnarski, John S. Griesenbeck, Norman C. Waters

**Affiliations:** Department of Bacterial and Parasitic Diseases, Armed Forces Research Institute of Medical Sciences, Bangkok, Thailand

**Keywords:** Exonuclease, Gene editing, Malaria, Piperaquine resistance, *Plasmodium falciparum*

## Abstract

**Background:**

The rise in *Plasmodium falciparum* resistance to dihydroartemisinin–piperaquine (DHA–PPQ) treatment has been documented in the Greater Mekong Subregion with associations with mutations in the *P. falciparum* chloroquine resistance transporter (*pfcrt*) and plasmepsin 2 (*pfpm2*) genes. However, it is unclear whether other genes also play a role with PPQ resistance, such as the E415G mutation in the exonuclease (*pfexo*) gene. The aim of this study was to investigate the role of this mutation in PPQ resistance by generating transgenic parasites expressing the *pfexo*-E415G mutant allele.

**Methods:**

Transgenic parasite clones carrying the E415G mutation in PfEXO of the B5 isolate were derived by CRISPR-Cas9 gene editing and verified using PCR and gene sequencing. Polymorphisms of *pfkelch-13*, *pfcrt*, and *pfexo* were examined by PCR while the copy number variations of *pfpm2* were examined by both relative quantitative real-time PCR and the duplication breakpoint assay. Drug sensitivity against a panel of antimalarials, the ring-stage survival assay (RSA), the PPQ survival assay (PSA), and bimodal dose-response curves were used to evaluate antimalarial susceptibility.

**Results:**

The transgenic line, B5-*rexo*-E415G-B8, was successfully generated. The PPQ-IC_90_, %PPQ survival, and the bimodal dose-response clearly showed that E415G mutation in PfEXO of B5 isolate remained fully susceptible to PPQ. Furthermore, growth assays demonstrated that the engineered parasites grew slightly faster than the unmodified parental isolates whereas *P. falciparum* isolates harbouring *pfkelch-13*, *pfcrt*, and *pfexo* mutations with multiple copies of *pfpm2* grew much more slowly.

**Conclusions:**

Insertion of the E415G mutation in PfEXO did not lead to increased PPQ-IC_90_ and %PPQ survival, suggesting that this mutation alone may not be associated with PPQ resistance, but could still be an important marker if used in conjunction with other markers for monitoring PPQ-resistant parasites. The results also highlight the importance of monitoring and evaluating suspected genetic mutations with regard to parasite fitness and resistance.

**Supplementary information:**

The online version contains supplementary material available at 10.1186/s12936-022-04148-z.

## Background

Artemisinin-based combination therapy (ACT) remains the recommended first-line antimalarial therapy for uncomplicated *Plasmodium falciparum* infections in almost all endemic countries [1]. ACT consists of two drugs: a semisynthetic artemisinin (ART) derivative and a longer-lasting partner drug. Currently, six artemisinin-based combinations are available including artemether + lumefantrine (AL), artesunate + amodiaquine (AS + AQ), artesunate + mefloquine (AS + MQ), artesunate + sulfadoxine–pyrimethamine (AS + SP), dihydroartemisinin + piperaquine (DHA + PPQ) and most recently artesunate + pyronaridine (AS + PND) [1]. However, the effectiveness of ACT is threatened by the emergence of parasites with decreased susceptibility to the ART derivatives and/or resistance to ACT partner drugs [2–4]. Soon after DHA–PPQ was recommended by the World Health Organization (WHO) as a first-line therapy for uncomplicated *P. falciparum* malaria [5], the emergence of DHA–PPQ resistance was reported within the Greater Mekong subregion including Cambodia [6–9], Vietnam [10], and more recently in Thailand [11].

ART resistance is characterized by the presence of mutations in the *Pfkelch13* (*pfk13*) propeller domain gene, which is associated with delayed in vivo parasite clearance times [2, 3] and with increased survival in the in vitro ring-stage survival assay (RSA) [12]. There are three molecular marker candidates for PPQ resistance: multiple copies of *P. falciparum* plasmepsin 2 (*pfpm2*) (PF3D7_1408000) [13–16], novel mutations in *P. falciparum* chloroquine resistance transporter (PfCRT) (PF3D7_0709000) [17–20], and a single mutation in *P. falciparum* exonuclease (PfEXO) (PF3D7_1362500) [13, 14, 21]. There have been eight previous publications reporting on the E415G mutation in the *pfexo* gene [13, 22–28]. In addition to the amplification of *pfpm2* and *pfpm3*, Amato et al. [13] identified a non-synonymous single nucleotide polymorphism (SNP) E415G substitution on an exonuclease-encoding gene from recrudescence isolates from DHA–PPQ treatment failures in Cambodia. The prevalence of DHA–PPQ treatment failures was at 2% in Ratanakiri, 16% in Preah Vihear, and 46% in Pursat. In Vietnam, neither PfEXO-E415G mutations nor *pfpm2* amplification was observed in parasites from south-central Vietnam between 2015 and 2016 [27], but *P. falciparum* samples collected from 2018 to 2019 in Vietnam’s Central Highlands [25] were found to harbour the PfEXO-E415G mutation with a prevalence of 66.7% and 85.5% in Dak Nong and Dak Lak, respectively. Si et al. [26] have recently reported that no parasites from the China-Myanmar border carried PfEXO-E415G, novel PfCRT mutations, or *pfpm2* amplification, even though some parasites with higher PPQ survival assay (PSA) values were detected, indicating different mechanisms of reduced PPQ susceptibility. In Africa, Robert et al. [23] first investigated the presence of both the PfEXO-E415G mutation and multiple copies of *pfpm2* in Senegalese parasites; however, no isolated parasites carried both molecular markers. A similar trend was observed in Sudan where no PfEXO-E415G mutations were observed [28]. However, in Mali, of 214 *P. falciparum* samples from Dangassa, two isolates exhibited the PfEXO-E415G mutation [22].

While the implication of multiple copies of *pfpm2* and novel PfCRT mutations on PPQ resistance have been elucidated [19, 20, 29], and the PPQ-resistance phenotype can be observed in the presence of novel PfCRT mutations with the PfEXO-E415G mutation [24], there is still a lack of confirmation as to whether the PfEXO-E415G mutation alone causes PPQ-resistance. To investigate whether the PfEXO-E415G is directly associated with PPQ resistance, in this study, CRISPR-Cas9 genome editing was used to generate *P. falciparum* Cambodian parasites harbouring PfEXO-E415G. The in vitro susceptibility to piperaquine and other frontline antimalarials of these gene-edited parasites was determined and compared to those of validated PPQ-resistant *P. falciparum* clinical isolates of Cambodian origin.

## Methods

### *pfexo* gene and amino acid prediction

The nucleotide sequence of *pfexo* from 3D7 was obtained from PlasmoDB with the accession number PF3D7_1362500. The *P. falciparum* B5 line was obtained from the cloning of a Cambodian *P. falciparum* isolate [24]. This isolate is ART- and PPQ-sensitive, but CQ-resistant. The full-length *pfexo* gene of B5 was amplified from *P. falciparum* B5 genomic DNA using polymerase chain reaction (PCR) with primers PA_exon1 and Screening_3’UTR_Rev (Additional file 1: Table S1). The encoded amino acid sequences and the nucleotide sequences were aligned using Clustal Omega [30]. The signal peptide region was predicted using Signa-P.5.0 [31] and the InterPro program was used for protein family classification [32].

### *Plasmodium falciparum* culture

Asexual blood-stages of *P. falciparum* were maintained in fresh human erythrocytes (O^+^) in RPMI1640 (Sigma-Aldrich, USA) supplemented with 5.94 gL^−1^ HEPES, 2.1 gL^−1^ sodium bicarbonate, 0.1 gL^−1^ gentamycin sulphate, 0.5% (w/v) Albumax II, 4 gL^−1^ dextrose 0.05 gL^−1^ hypoxanthine, and 10–15% of human serum (complete medium) [33]. Human blood products (erythrocytes and serum) were obtained from the Thai Red Cross. Culture flasks were gassed with 5% CO_2_, 5% O_2_, 90% N_2_ gas and incubated at 37 °C. For synchronisation, mature schizont stage parasites were isolated on cushions of 75% (v/v) isotonic Percoll cushion (GE Healthcare Life Science) as previously described [34, 35] whereas enrichment for ring stages following invasion was performed using 5% (w/v) d-sorbitol [36] The final ring cultures were washed and returned to culture or used as required.

### Construction of plasmid constructs to genetically modify *P. falciparum*

PCR amplicons used in plasmid cloning were generated using Fusion, High Fidelity DNA polymerase (New England Biolabs) and purified using Qiagen PCR purification or Qiagen Gel extraction kits. For diagnostic PCR amplification, GoTaq (Promega) DNA master mix was used. All constructed plasmids were sequenced to verify authenticity (Biobasic, Canada). For parasite genomic DNA extraction, total cell pellets were first treated with 0.15% saponin in PBS for 10 min, then washed with PBS before DNA was extracted using a DNeasy Blood & Tissue Kit (Qiagen).

#### Construct for donor plasmids

A fragment of *exo* sequence with E415G mutation (1441 bp) was commercially synthesised (GenScript, USA), comprising a stretch of native *P. falciparum* 3D7 *exo* sequence (covering residues Lys283, *exo* intron, and Asn408) followed by a stretch of recodonized *exo* gene sequence encoding the 3D7 amino acid residues Met409 to Glu514 with the point mutation at residue 415 from Glu to Gly, but using a different codon usage, and finally a stretch of native 3D7 *exo* sequence covering amino acid residues 515 to the stop codon. This fragment was cloned into pUC57 by *EcoRV* generated pUC57-Exo-E415G plasmid. To generate a donor plasmid to introduce Glu415Glu wild-type, pUC57-Exo-E415E plasmid was derived from pUC57-Exo-E415G by Q5 site-directed mutagenesis (New England Biolabs) to alter the codon encoding at amino acid position 415 from GGC to GAA primers Q5SDM_G415E_F and Q5SDM_G415E_R were used (Additional file 1: Table S1).

#### Construct for CRISPR-Cas9 plasmids

pDC2-Cas9-hDHFR-yFCU, containing a Cas9 expression cassette and the drug selection marker human dihydrofolate reductase (*hdhfr*) [37] was used as a template vector to generate pDC2-Cas9-bsd-yFCU. Since the *P. falciparum* B5 strain is resistant to pyrimethamine, the *hdhfr* gene was replaced with the *blasticidin* gene (*bsd*). The *bsd* gene (399 bp) was amplified from PkpSKIP_Pk47 plasmid [38] using primers bsd_NcoI_F and bsd_SacII_R (Additional file 1: Table S1) and was cloned into the NcoI/SacII-digested pDC2-Cas9-hDHFR-yFCU, giving rise to pDC2-Cas9-bsd-yFCU.

Guide RNA sequences specific for targeting at amino acid position 415 were identified using Benchling (https://www.benchling.com/crispr/) (Additional file 1: Fig. S1). A pair of complementary oligonucleotides (sgE415G-1F and sgE415G-1R) corresponding to the 19 nucleotides adjacent to the identified PAM sequences were phosphorylated using T4 polynucleotide kinase, annealed and ligated into pDC2-Cas9-bsd-yFCU predigested with BbsI, resulting in the guide vector pGuide1.4-bsd.

### Generation of *P. falciparum* lines expressing *exo*-E415G mutant

The donor plasmid pUC57-Exo-E415G was linearized with ScaI prior to electroporation. Percoll-enriched mature schizonts of *P. falciparum* B5 were electroporated with 20 µg of pGuide1.4-bsd and 60 µg of linearized pUC57-Exo-E415G using either Amaxa P3 primary cell 4D Nucleofector X Kit L (Lonza) or Amaxa Basic Parasite Nucleofector Starter Kit as described [39, 40]. Twenty-four hours post-transfection, the electroporated parasites were treated with 5.45 µM blasticidin-*S*-hydrochloride (Sigma-Aldrich, USA) to select for transfectants harbouring pGuide1.4-bsd before returning the cultures to medium without drug. Detection of the *exo*-E415G modified locus was carried out by diagnostic PCR using primer pairs ExonI_K283_F and Screen_WT_Rev_V616, ExonI_K283_F and Recodon_R, and Recodon_F and Screen_3′UTR_R. The wild-type *pfexo* locus was detected by diagnostic PCR using primers ExonI_K283_F and Screen_WT_Rev_V616, with a PCR product of 766 bp. Transgenic parasite clones were obtained by limiting dilution cloning by plating a calculated 0.3 parasite per well (200 µL and 1% haematocrit) in flat-bottomed 96-well microplate wells as described [41]. Wells containing single plaques were identified after 10–14 days using an inverted microscope and the parasites subsequently expanded into round-bottomed wells for further analysis. Transgenic parasite clones were finally checked by diagnostic PCR for integration and modification of the endogenous *pfexo*^E415G^ gene. A *pfexo*^E415E^ transgenic line expressing the wild-type Glu415 codon was generated in the same manner of that for a *pfexo*^E415G^ transgenic line by transfecting *P. falciparum* B5 with 20 µg of pGuide1.4-bsd and 60 µg of linearized pUC57-Exo-E415E. Once established, all transgenic clones were maintained in medium without any drug.

### *pfk13*, *pfcrt*, and *pfexo* genotyping

Master Cycler Nexus Gradient (Eppendorf) was employed to evaluate the propeller domain of the *P. falciparum* kelch13 (*pfk13*) (amino acid residues 442–727) [42, 43], *P. falciparum* exonuclease (*pfexo*) SNP at a codon corresponding to the amino acid position 415 [13], and the *P. falciparum* chloroquine resistant transporter (*pfcrt*) SNPs at codons corresponding to amino acid positions 93, 97, 145, 218, 343, 350, and 353 [19, 44]. Primers used to identify *pfk13*, *pfexo*, and *pfcrt* SNPs are shown in Additional file 1: Table S1. *Plasmodium falciparum* reference DNAs from 3D7 and W2 clones (Malaria Research & Reference Reagent Resource, Manassas, VA) were used as positive controls, and all samples were performed in duplicate.

### Plasmepsin (*pfpm*) 1, 2 and 3 copy number variation assay

To determine copy numbers of *pfpm1* (PF3D7_1407900), *pfpm2* (PF3D7_1408000), and *pfpm3* (PF3D7_1408100) gene, real-time quantitative PCR (qPCR) was performed on genomic DNA as previously described [29]. The amplification reactions were performed according to Luna® Universal qPCR master mix kit (New England Biolabs) with 200 nM of each forward and reverse primer (Additional file 1: Table S1) and 2 ng of DNA template using Rotor-Gene Q (QIAGEN, Valencia, CA). For the housekeeping gene, *β-tubulin* (PF3D7_1008700), *β-tubulin* forward and reverse primers were designed and used as a reference control for all experiments with the same validated PCR conditions as target primers. *P. falciparum* 3D7 was used as a reference clone. All samples including the references clones were performed in triplicate. The average copy number values for each gene were calculated using 2^−ΔΔCt^ method where ΔΔCt is [Ct_*pfpm*_ − Ct_*pf β-tubulin*_]_sample_ − [Ct_*pfpm2*_ − Ct_*pf β-tubulin*_]_3D7_. Parasites with copy number greater than 1.6 copies for *pfpm2* and *pfpm2* [15, 16] were interpreted to contain multiple copies.

### Plasmepsin 2/3 (*pfpm*2/3)duplication breakpoint PCR assay

The *pfpm2/3* breakpoint PCR assay was performed as previously described [45]. Three pairs of primers (Additional file 1: Table S1) were used in this assay. Primers AF_for and AR_rev amplified a 623 bp product surrounding the breakpoint located at the 3′ end of *pfpm1*. Primers BF_for and BR_rev amplified a 484 bp product surrounding the breakpoint at the 3′ end of *pfpm3.* Primers BF_for and AR_rev amplified the junction between the breakpoint, producing a 497 bp product in parasite isolates with *pfpm2/3* amplifications. A *pfpm2/3* single copy isolate is not expected to have the PCR product with these primers. One copy isolate was only noted when the control primer sets amplified a product, and the duplication PCR was negative. Two or more copies were annotated as > 1 copy of *pfpm2/3* only when both the control and duplication primer sets generated a product. PCR reactions contained 12.5 µL GoTaq® Green Master Mix (Promega), 1 µL of each primer (10 µM stocks), 3 µL of DNA up to 25 µL final volume with water. PCR conditions were as follow: 95 °C for 2 min, followed by 30 cycles of 95 °C for 45 s, 50 °C for 30 s, 72 °C for 1 min, followed by a 5-min extension at 72 °C.

### In vitro drug susceptibility

Drug susceptibility testing used HRP-2 ELISA to measure 50% or 90% inhibitory concentration (IC_50_ and IC_90_) performed as previously published [46, 47]. In vitro drug susceptibility testing was carried out for control reference clones (W2, D6, C2B) (Malaria Research & Reference Reagent Resource, Manassas, Vermont, USA) as described previously [48]. IC_50_s and IC_90_s were estimated by nonlinear regression analysis using GraphPad Prism version 6.0 program. Samples having poor growth rate, as perceived by obtaining an OD ratio of < 1.7 between the no-drug test wells and the maximum tested drug concentration, were excluded from data analysis.

### Bimodal dose response curve

To determine a bimodal-dose response curve, the PPQ concentration (2.44 to 100,000 ng/mL) and the dilution series were increased from 8 to 24 points, according to previously published reports [16, 24]. Culture-adapted clinical isolates or engineered parasites were prepared in the similar manner as in in vitro drug susceptibility testing. The synchronized rings were grown for 72 h in the presence of different concentrations of PPQ (24-point dilution) in 96-well plates at 1.5% haematocrit, 0.5% starting parasitaemia in 0.5% Albumax RPMI 1640. Growth at 72 h was measured by HRP-2 ELISA. Assays were carried out in three biological replicates and the control reference clone W2 was tested along with each culture-adapted clinical isolate. The area under the curve (AUC) for the dose response curve at 0.01–100 µM was calculated using GraphPad Prism 6.0.

### Ring-stage survival assay (RSA)

In vitro RSA_0–3 h_ was performed on 0–3 h post-invasion rings obtained from selected culture-adapted clinical isolates following published methods [12] with slight modifications. Briefly, parasite cultures were tightly synchronized using 5% (w/v) d-sorbitol and 75% Percoll to obtain 0 to 3-h post-invasion rings which were adjusted to 0.5–1% starting parasitaemia with a 2% haematocrit in culture media (0.5% Albumax RPMI 1640 with 2.5% AB serum) and cultured in a 48-well microplate with 700 nM DHA and 0.1% DMSO in separate wells for growth control. The culture plate was then incubated for 6 h at 37 °C in modular incubator chambers and gassed with 5% CO_2_, 5% O_2_ and 90% N_2_ gas. Cells were then washed once, resuspended in drug-free medium, and cultured further for 66 h. Susceptibility to DHA was assessed microscopically on thin films by estimating the percentage of viable parasites, relative to control (% survival rate). For the controls, the RSA_0–3 h_ was also performed on *P. falciparum* reference clones W2 (ART-sensitive control), IPC-4884 and IPC-5202 (BEI Resources, NIAID, NIH, USA) as ART-resistant control lines. A survival rate > 1% was deemed resistant for RSA.

### Piperaquine survival assay (PSA)

PSA_0–3 h_ was performed on culture-adapted clinical isolates with 0–3-h ring-stage parasite cultures following a previously published method [17]. Briefly, parasite cultures were tightly synchronized using 5% (w/v) d-sorbitol and 75% Percoll to obtain 0 to 3-h post-invasion. Synchronized ring parasites at 0.5–1% starting parasitaemia and 2% haematocrit were incubated with 200 nM PPQ or 0.5% lactic acid in water at 37 °C for 48 h in a 48-well microplate. The cultures were then washed once, resuspended in drug-free medium, and cultured further for 24 h. Susceptibility to PPQ was assessed microscopically on thin films by estimating the percentage of viable parasites in the similar manner as RSA. A survival > 10% was deemed resistant to PPQ.

### Growth assays

For longer-term replication assays, cultures were synchronized as described and resulting in ring stage cultures. The ring stage parasites were synchronized using 5% (w/v) d-sorbitol and parasitaemia levels were calculated, and cultures adjusted to 0.1% parasitaemia, 2% haematocrit in a final volume of 1 mL per well of a 12-well plate. Samples were then taken at t = 0, 24, 72, 120, and 168 h, fixed in 0.8% glutaraldehyde in PBS and stored at 4 °C for flow cytometry analysis. Culture media was replaced at 48, 71, and 120 h. Giemsa-stained thin films were also prepared as required for microscopic analysis.

### Flow cytometry for parasite quantification

Parasite samples were fixed in 0.8% glutaraldehyde in PBS and stored at 4 °C. Cells were prepared for analysis by staining with 2× SYBR Green I nucleic acid gel stain (Invitrogen, Thermo Fisher Scientific) for 30 min at 37 °C. Labelling was stopped with an equal volume of PBS and samples acquired using a CytoFlex (Beckman Coulter, USA) with CyEXpert software. Total RBC numbers were calculated using forward- and side-scatter while fluorescence was detected using the 530/630 blue detection laser. Fluorescence intensity was used to distinguish uninfected from infected RBCs, low fluorescence indicating uninfected cells and gating fixed accordingly. Data were analysed using FlowJo.

### Statistical analysis

Statistical analysis was performed using GraphPad Prism version 6.0 (GraphPad Software, Inc., San Diego, CA, USA). The difference of the data between groups was assessed by nonparametric Mann–Whitney U test, as appropriate. Statistical significance was defined as a P value of < 0.05.

## Results

### CRISPR-Cas9-mediated editing to introduce *pfexo*^E415G^ into *P. falciparum* parasites

The *pfexo* gene from *P. falciparum* 3D7 (PF3D7_1362500) consists of 2274 bp with 2 exons and 1 intron. Exon 1 comprises nucleotides 1-949, while the exon 2 starts from nucleotides 1082–2274 (Additional file 1: Fig. S1). PfEXO consists of 713 amino acids with a molecular mass of 86.7 kDa. The enzyme contains a peptide signal region (residues 1–70) and a 3′ to 5′ exonuclease domain (residues 445–618). The DNA sequence of the *pfexo* gene from *P. falciparum* B5 [24], the parental line for genome editing in this study, showed a single non-synonymous mutation, leading to a missense mutation from Lys to Asn at residue 614 (Additional file 1: Fig. S2).

**Fig. 1:**
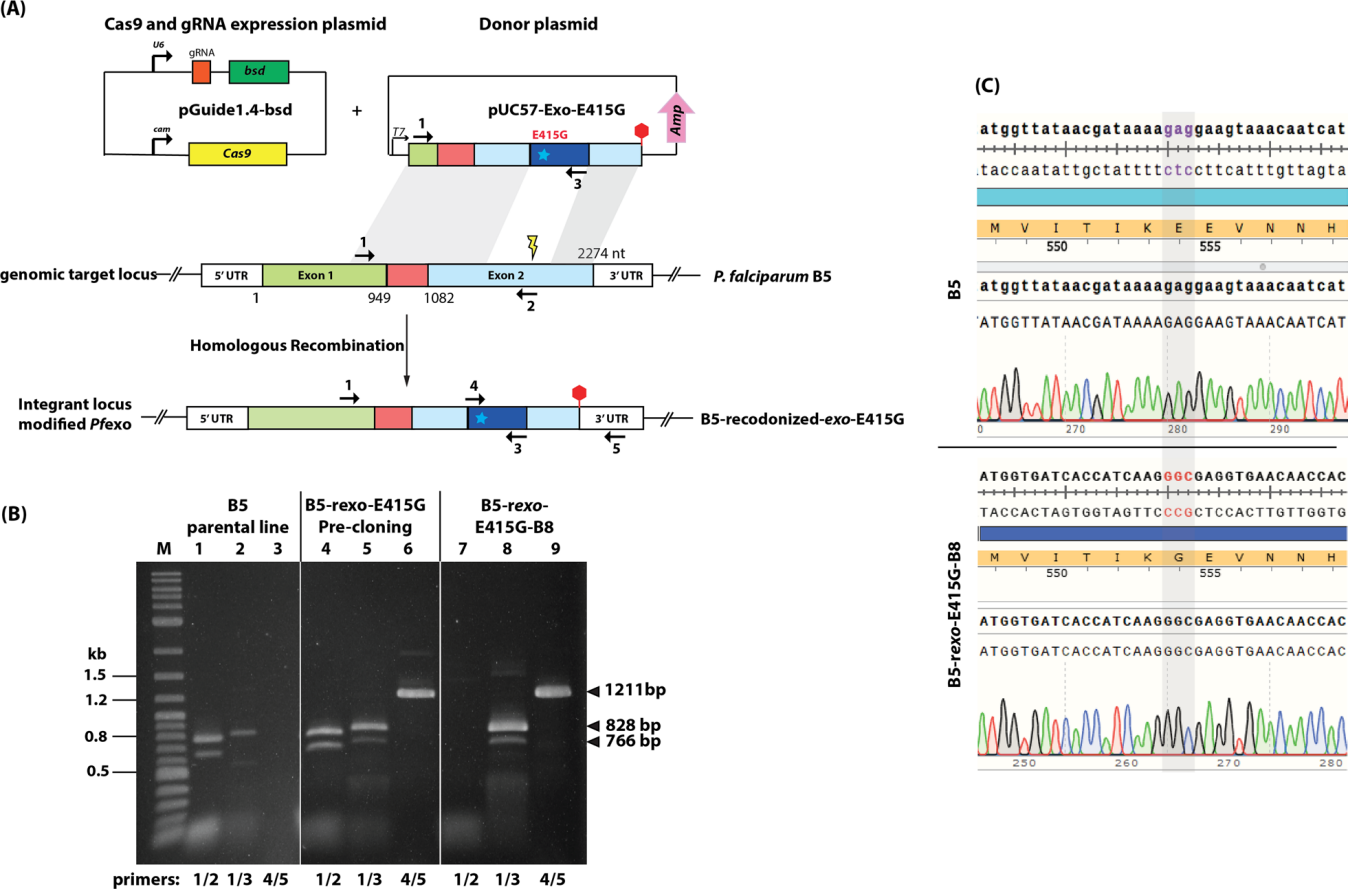
Gene editing of pfexo. (**A**) CRISPR-Cas9 editing was used to install the pfexoE415G variant codon into the endogenous locus. Using a donor plasmid and Cas9-aided homologous recombination, the region of the pfexo encoding Met409 to Glu514 was replaced with a recodonized pfexo gene (navy blue) with the point mutation at residue 415 (a cyan star) from Glu to Gly. Stop codon is represented as a red hexagon. Position of hybridisation of primers used for confirmation of the integration event by diagnostic PCR are shown as coloured arrows. A schematic of the co-transfected plasmid for expression of Cas9 nuclease and the guide RNAs is also shown. (**B**) Diagnostic PCR analysis of genomic DNA of the control parental B5 line, the transfected parasites prior to limiting dilution cloning and clone parasite lines expressing pfexoE415G (clone B8). Expected sizes of the various PCR amplicons are indicated on the right of the gel, whilst the left-hand lane contains double-stranded DNA ladder marker fragment (Quick-load Purple 1 kb Plus DNA ladder (New England Biolabs)). (**C**) Chromatograms of a region of exon 2 obtained from the PCR product amplified pfexo gene of B5 and B5-rexo-E415G-B8 lines. The codon “GAG” encoding Glu in B5 parasites is changed to “GGC” encoding Gly in B5-rexo-E415G-B8 clone

To evaluate the impact of the PfEXO-E415G mutation on parasite susceptibility to piperaquine and other front-line chemotherapies, CRISPR-Cas9 editing was used to introduce the E415G mutation into the native *pfexo* gene (Fig. 1A). A *pfexo*^E415G^ was efficiently installed onto the *P. falciparum* B5 background [24]. In parallel, a *pfexo*^E415E^ transgenic line expressing wild-type E415 codon in the context of recodonization abating the Cas9 protospacer adjacent motif (PAM) site was also attempted to rule out a role for silent mutations in drug susceptibility. Eleven transfections were performed to introduce *pfexo*^E415G^ mutation and 3 transfections for *pfexo*^E415E^ mutation but only three transfections provided revived parasites. Two of the three transfections gave rise to the modified *pfexo* gene, and one transfection did not provide the modified parasites. Successful modification of the *pfexo* gene in the transfected population following the introduction of the targeting vector was observed around 21 days after transfection and confirmed by diagnostic PCR (Fig. 1B, pre-cloning). Limiting dilution cloning of the modified parasites resulted in the isolation of parasite clone, B5-r*exo*-E415G-B8. It is noted that several clones obtained from the cloning did not contain the modified region of the *pfexo* gene. Modification of the native *pfexo* locus was then confirmed in the transgenic line by diagnostic PCR (Fig. 1B, B5-r*exo*-E415G-B8) and genomic sequences of transgenic lines (Fig. 1C). It is noted that the band of 828 bp appearing on lane 2 (Fig. 1B) arose from the non-specificity of primer 3 which can anneal to both parental and transgenic parasite lines. Unfortunately, the *pfexo*^E415E^ transgenic line could not be obtained from this study.

### Molecular genotyping of modified *pfexo* transgenic lines and clinical isolates with PfEXO-E415G mutation

The genotypic profiling for piperaquine and artemisinin molecular markers is outlined in Table 1. In addition to the reference clones and engineered parasite lines, two Cambodian isolates from a previous *P. falciparum* clinical efficacy study (ASAP-21 and ASAP-168) [49] were included as they harboured PfEXO-E415G with and without novel PfCRT mutations. As expected, the transgenic B5-rexo-E415G-B8 line carried a similar genotypic profile as the parental B5 line except for the E415G mutation in the *pfexo* gene. Regarding the two clinical isolates, both carried the PfK13-C580Y and PfEXO-E415G mutations. The ASAP-21 isolate harboured the novel PfCRT*-*F145I mutation while ASAP-168 did not. Genetic studies have identified copy number variations in the *pfpm2* and *pfpm3* genes that associate with clinical and in vitro PPQ resistance [13, 15]. For *pfpm2/3* amplification, the putative breakpoints lie near the 3′ end of both *pfpm1* and *pfpm3* so each amplification produces an intact extra copy of *pfpm2* together with a new chimeric pfpm3 with its 3′ end replaced by the 3′ end of *pfpm1* (Additional file 1: Fig. S3). This result showed that the copy number variation determined by qPCR assay showed that all parasite lines harboured a single copy of *pfpm1*. Two methods were then carried out to determine the copy number variation of the *pfpm2* and *pfpm3* gene: the SYBR-green qPCR and breakpoint assays [45]. By using the qPCR assay, ASAP-21 was found to contain multiple copies of *pfpm*2 and ASAP-168 carried a single copy of *pfpm*2, whereas B5 parental line and transgenic B5*-*r*exo*-E415G-B8 were found to have multiple copies of *pfpm3*. On the contrary, both ASAP-21 and ASAP-168 isolates were positive for *pfpm*2/3 amplification (>1 copy) while B5 and B5*-*r*exo*-E415G-B8 lines showed no *pfpm2/3* amplification as detected by the breakpoint assay (Additional file 1: Fig. S3). The non-concordant detection between the two methods were also reported by Ansbro et al. [45] and it was suggested that the breakpoint PCR assay is more sensitive than the qPCR assay for detecting minor clones containing the duplication in field isolates. Therefore, in this study, ASAP-21 and ASAP-168 isolates were treated as having multiple copies of *pfpm*2/3 amplification, while B5 and B5*-*r*exo*-E415G-B8 lines contained a single copy of *pfpm2* and *pfpm3.*

**Table 1:** Molecular genotyping of *pfexo*-modified, parental and clinical *P. falciparum* parasites

Sample	PfK13 C580Y	PfEXO E415G	PfCRT							Copy number variation			*pfpm2/3* break point
			T93S	H97Y	F145I	I218F	M343L	C350R	G353V	*pfpm1*	*pfpm2*	*pfpm3*	
3D7	C	E	T	H	F	I	M	C	G	1.00	1.00	1.00	1
B5	C	E	T	H	F	I	M	C	G	1.37	0.93	**1.99**	1
B5*-*r*exo*-E415G-B8	C	**G**	T	H	F	I	M	C	G	1.19	0.74	**2.08**	1
ASAP-21	**Y**	**G**	T	H	**I**	I	M	C	G	1.25	**2.31**	**2.60**	**> 1**
ASAP-168	**Y**	**G**	T	H	F	I	M	C	G	1.18	1.11	**1.88**	**> 1**

Underline and bold letters indicate either mutations or multiple copy number. A cut-off copy number of 1.6 is used to define *pfpm2 and pfpm3* multiple copy number

### Drug sensitivity and survival assay of modified *pfexo* transgenic lines and clinical isolates

The transgenic parasites expressing E415G mutant of PfEXO and the clinical isolates were tested for their PPQ susceptibility (Table 2). No significant difference in IC_50_ values of B5-r*exo*-E415G-B8 and ASAP-168 parasites compared to B5 parasites was observed, while PPQ-IC_50_ value of ASAP-21 parasites was significantly higher than that of B5 parasites. The same trend was observed for PPQ-IC_90_ values in that there was no significant difference in the IC_90_ values for B5-r*exo*-E415G-B8 and ASAP-168 parasites compared to B5 parasites. The PPQ-IC_90_ of ASAP-21 exceeded the highest PPQ concentration used in this study, so the IC_90_ could not be reported.

**Table 2:** Piperaquine (PPQ) susceptibility (mean ± SD)

Sample	PPQ-IC_50_ (nM)	PPQ-IC_90_ (nM)
B5	112 ± 54	172 ± 9
B5*-*r*exo*-E415G-B8	185 ± 54^1^	243 ± 22^1^
ASAP-21	5207 ± 690^2^	N.D.
ASAP-168	111 ± 17^1^	312 ± 37^1^

^1^No significant difference (*P* value > 0.05) compared to B5 as tested by Mann–Whitney U Test

^2^Significant difference (*P* value = 0.0167) between ASAP-21 and B5 as tested by Mann–Whitney U Test

N.D. for not determined

To evaluate if the modified *pfexo* transgenic parasites and clinical isolates with PfEXO-E415G mutation have cross-resistance, parasite drug sensitivity was also assessed against a panel of antimalarial drugs (Table 3). No significant difference in IC_50_ values between B5 and B5-rexo-E415G-B8 parasites were observed for most of antimalarial drugs except for atovaquone (ATG) and proguanil (PG), where higher IC_50_ values were detected in comparison with the B5 parental line. The ASAP-21 parasite had significantly lower IC_50_ values for quinine (QN), chloroquine (CQ), atovaquone (ATQ) and doxycycline (DOX) but higher IC_50_ value for pyronaridine (PND) compared to the B5 parental line, while the ASAP-168 parasite had  increased drug sensitivity against dihydroartemisinin (DHA), mefloquine (MQ), QN, and ATQ. The reduced IC_50_ values of QN and ATQ were observed in both ASAP-21 and ASAP-168 parasites compared to the B5 parental line.

**Table 3:** In vitro susceptibility of *P. falciparum*, parental (B5), *pfexo*-modified (B5-rexo-E415G-B8) and adapted clinical parasites to dihydroartemisinin (DHA), artesunate (AS), mefloquine (MQ), quinine (QN), chloroquine (CQ), atovaquone (ATQ), lumefantrine (LUM), doxycycline (DOX), tafenoquine (TQ), cycloguanil (CYC), primaquine (PQ), proguanil (PG), and pyronaridine (PND). (mean ± SD, nM)

Drug	IC_50_ (nM)				
	B5	B5***-***r *exo*-E415G-B8	ASAP-21	ASAP-168	*P*-value
DHA	9 ± 4	14 ± 6	5 ± 0.2	2 ± 1	0.121^3^
AS	7 ± 5	4 ± 1	3 ± 0.4	3 ± 1	–
MQ	161 ± 58	82 ± 37	61 ± 23	50 ± 20	0.048^3^
QN	546 ± 180	564 ± 165	71 ± 3	138 ± 32	0.017^2,3^
CQ	562 ± 218	416 ± 219	64 ± 12	256 ± 59	0.017^2^
ATQ	48 ± 38	114 ± 1	5 ± 0.6	4 ± 1	0.017^1,2,3^
LUM	10 ± 8	4 ± 1	2 ± 0.2	3 ± 1	–
DOX	13,523 ± 2337	14,649 ± 1071	8527 ± 349	10,920 ± 1061	0.024^2^
TQ	263 ± 143	315 ± 45	189 ± 13	274 ± 554	–
CYC	2063 ± 2703	705 ± 15	7183 ± 388	N.D.	–
PQ	3094 ± 2015	6387 ± 218	5720 ± 1160	2458 ± 61	–
PG	12,522 ± 7247	23,816 ± 3786	2114 ± 243	3643 ± 473	0.033^1^
PND	28 ± 16	25 ± 4	50 ± 2	12 ± 6	0.033^2^

*P*-value calculated by Mann–Whitney U Test

^1,2,3^Significant differences between data from B5-rexo-E415G-B8, ASAP-21, and ASAP-168 compared to B5 line, respectively

N.D. for not determined

**Fig. 2:**
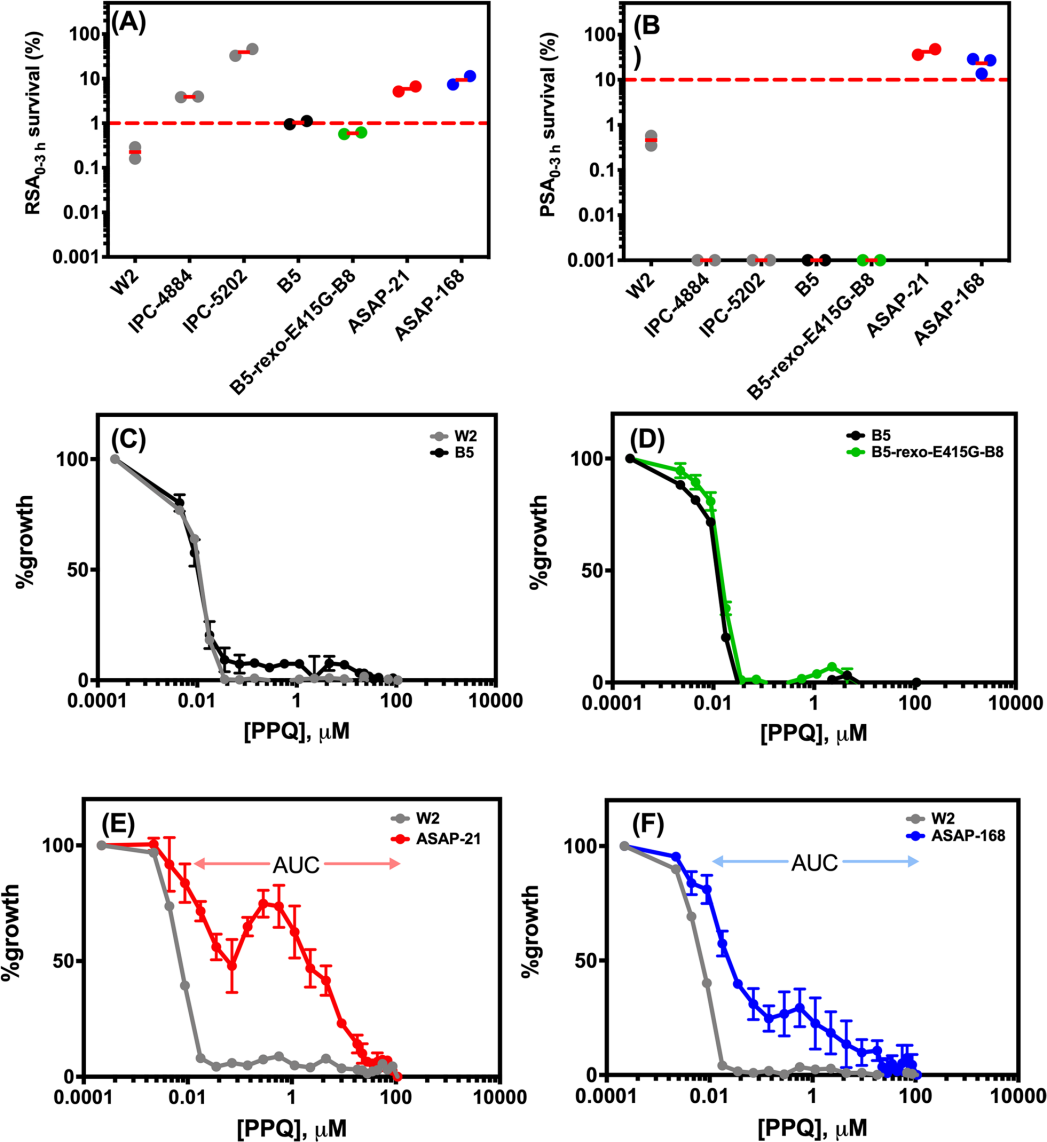
Phenotypic analysis of pfexo transgenic lines and clinical isolates. (**A**-**B**) In vitro RSA_0-3h_ and PSA_0-3h_  survival rates, respectively, for standard laboratory-adapted clones (W2 for an ART-sensitive control, IPC-4884 and IPC-5202 for ART-resistance control), B5 (parental line, B5-rexo-E415G-B8 (modified pfexo transgenic line) and culture-adapted clinical isolates (ASAP-21 and ASAP-168). The dashed line represents the 1% survival rate cut-off that differentiates ART-resistance (≥ 1%) from ART-sensitive (< 1%) parasites in RSAs or the 10% survival rate cut-off that distinguishes PPQ-resistance (≥ 10%) from PPQ-sensitive (< 10%) parasites in PSAs. Two independent biological replicates were performed. Zero values of % survival rate were plotted as 0.001 % in logarithmic scale. (**C**-**F**) PPQ-bimodal dose-response curves of B5, B8, ASAP-21, and ASAP-168, respectively. Increasing the starting concentration and number of data points (24 points) for HRP2 ELISA dose-response curve provided a bimodal distribution of parasite response to PPQ exposure for PPQ-resistant parasites. ASAP-21 and ASAP-168 shows a second peak in PPQ concentration 0.01- 100 µM. The concentration range used to calculate the area under the curve (AUC) is indicated. Data are shown as mean values from three biological replicates with S.D

To gain a better understanding of the ART and PPQ resistance phenotypes, the survival assay (RSA_0–3 h_ and PSA_0–3 h_) and PPQ-bimodal dose response curve were performed (Fig. 2). For RSA_0–3 h_ (Fig. 2A), *P. falciparum* W2 was used as a control of ART-sensitive parasites, while IPC-4884 and IPC-5202 parasites were controls for ART-resistant parasites. The ASAP-21 and ASAP-168 parasites, containing the PfK13-C580Y mutation, exhibited % RSA survival rate of greater than 1, while B5 and B5-rexo-E415G-B8 parasites, carrying wild-type PfK13, exhibited % RSA survival rate of less than 1, clearly validating the correlation between PfK13-C580Y mutation and ART resistance. The % PSA survival rate of the modified *pfexo* transgenic lines and clinical isolates were next examined (Fig. 2B). PPQ-sensitive parasites *P. falciparum* W2, IPC-4884 and IPC-5202 were used as controls. The transgenic B5-r*exo*-E415G-B8 parasites harbouring merely PfEXO-E415G mutation had a % PSA survival rate of less than 10 (a cut-off for PPQ resistance), similar to that of the parental B5 parasites. This suggested that the presence of PfEXO-E415G mutation alone could not confer the PPQ resistance phenotype. The ASAP-21 and ASAP-168 parasites, containing the PfEXO-E415G mutation in combination with either PfCRT mutation or multiple copies of *pfpm2*, exhibited a % PSA survival rate higher than 10, indicative of PPQ-resistance.

**Fig. 3:**
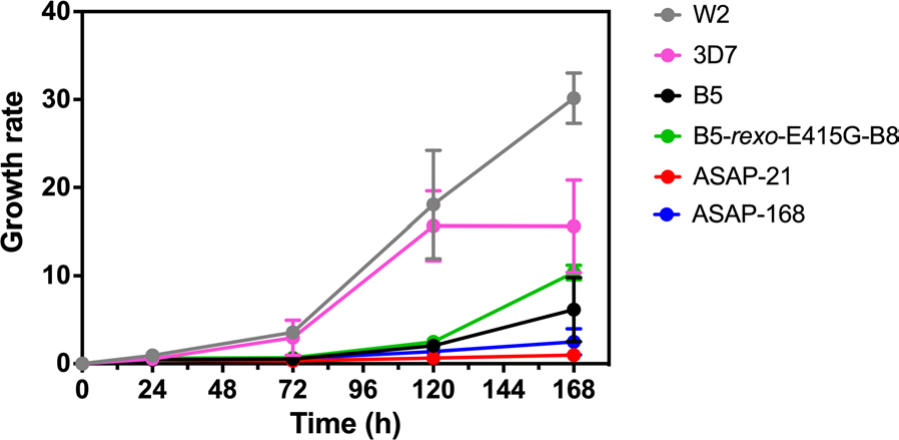
Analysis of growth rates. P. falciparum parasites with PfEXO wild-type (W2, 3D7 and B5), PfEXO-E415G transgenic line (B5-rexo-E415G-B8), and clinical isolates with the combination of PfEXO-E415G and multiple pfpm2 copies (ASAP-21 and ASAP-168) were synchronized (initiated at 0.1% parasitaemia ring stage, 2% haematocrit) and followed for 168 hours. Nucleic acids were stained with SYBR Green I and parasitaemia assessed by flow cytometry. Raw values were corrected, and data represent mean ± S.D. from 3 independent experiments

In addition to PSA_0–3 h_, PPQ-resistant parasites have been reported to exhibit a bimodal dose-response curve with a second peak between 0.01 and 100 µM and that the AUC correlates with the degree of PPQ resistance [16]. B5 and B5-r*exo*-E415G-B8 parasites did not exhibit the bimodal dose-response curve, confirming the PPQ-sensitive phenotype, whereas both ASAP-21 and ASAP-168 parasites clearly showed the bimodal dose-response curve with the AUC of 1029 and 596, respectively (Fig. 3C–F). The AUC of these parasites was in good agreement with both IC_50_ and IC_90_ values in that ASAP-21 parasites are more resistant to PPQ than ASAP-168 parasites.

### Growth assay

To assess whether the introduction of E415G mutation into PfEXO affects the parasite fitness, growth assays of B5-r*exo*-E415G-B8 parasites was carried out in comparison with W2, 3D7, B5 and the other two culture adapted clinical isolates (ASAP-21 and ASAP-168) (Fig. 3). Parasite lines originating from Cambodia (i.e., B5, B5-r*exo*-E415G-B8, ASAP-21 and ASAP-168) grew much more slowly than W2 (an Indochina clone exhibiting CQ resistance) and 3D7 (African origin) lines. Parasite B5-r*exo*-E415G-B8, harbouring *pfexo*-E415G mutation, seemed to grow slightly faster than its parental line B5. In the presence of PfK13-C580Y, PfEXO-E415G, novel PfCRT*-*F145I mutations and multiple copy number of *pfpm2*, ASAP-21 parasite had a severely retarded growth rate. ASAP-168, having the similar genetic background to ASAP-21 but without the PfCRT*-*F145I mutation, grew marginally better than ASAP-21 parasites. This observation implies an important contribution of the strain background toward parasite fitness, especially when PfK13-C580Y, PfCRT*-*F145I mutations and multiple copy number of *pfpm2* were present.

## Discussion

Validated molecular markers, such as *pfk13* for ART resistance, have been widely employed as identification and prediction tools for the emergence of drug-resistant *P. falciparum* [50]. While a molecular marker of PPQ resistant *P. falciparum* malaria has been reported [13, 15, 16, 19, 20, 29], there is still a pressing need for validating potential new or additive molecular markers to determine effects on PPQ as a partner drug in ACT. This study found that the E415G mutation in the gene *pfexo* cannot alone reduce PPQ susceptibility neither improve parasite survival rates that are characteristic of PPQ resistant isolates circulating in the Greater Mekong Subregion [17].

Exonucleases are essential to genome stability, catalysing the removal of a single nucleotide monophosphate (dNMP) from the end of one strand of DNA and acting as a proof-reader during DNA replication [51]. Exonucleases are highly conserved and can be classified into families based on both sequence and functional homology, including the 5′–3′ exo C-terminal domain (CTD) superfamily, the RNAaseH domain superfamily i.e., DnaQ-like family and other 3′–5′ exonucleases. The *pfexo* gene from *P. falciparum* (PF3D7_1362500) encodes a protein with a mass of 86.7 kDa with a 3′–5′ exonuclease domain at the C-terminus. In this study, the *P. falciparum* B5 line used for genome editing had one mutation (K614N) in exonuclease, located in the 3′–5′ exonuclease domain. Since no in vivo relevance of this mutation has yet been proved in this study, it is suggested that the prevalence of this mutation should be determined. Nucleases may be partially or fully redundant, depending on the pathway, and such redundancy might complement functional losses. Zhang et al. [52] used bioinformatics to predict eight putative RNA exosome-associated proteins in the *P. falciparum* genome, including exoribonuclease functional domain-containing proteins Dis3 and Rrp6, the latter of which was found in PfEXO of this study.

CRISPR/Cas9 approaches have transformed the speed and scale with which *Plasmodium* genome editing can be achieved, and the approach has been employed to introduce point mutations into several *P. falciparum* genes [53]. In this study, instead of using *P. falciparum* 3D7 or Dd2 lines, the Cambodia adapted-B5 line [24] was employed with the hypothesis that the B5 line has a close genetic background to the currently circulating *P. falciparum* in Cambodia. It has been previously shown that different phenotypes were observed when using different parasite backgrounds. Targeted gene disruption of either *pfpm2* or *pfpm3* in the 3D7 genetic background caused a only slight decrease in PPQ susceptibility [54] and *pfpm2* and *pfpm3* overexpression in 3D7 did not alter the sensitivity of *P. falciparum* to PPQ [55]. However, when *P. falciparum* Dd2 parasites with copy number variation in *pfpm2* were generated [29], *pfpm2* amplification contributed to PPQ resistance, with a bimodal dose-response observed. In addition to a qPCR assay to detect *pfpm2* copy number variation, the breakpoint assay, as described by Imwong et al. [56], was carried out. It was found that in parasites with a *pfpm2/3* copy number above the cut-off of 1.52, 88% were confirmed to have *pfpm2/3* amplification by the breakpoint assay, while for those < 1.14 (the cut-off for a single copy number) and for an intermediate value between 1.14 and 1.52, the proportion of *pfpm2/3* amplified parasites using the breakpoint SNP were 4% and 38%, respectively. In this study, the ASAP-168 parasite was found to have *pfpm2* copy number variation of 1.11, lower than the cut-off for a single copy number, but the breakpoint assay confirmed the gene duplication of *pfpm2*. As for *pfpm3* copy number variation, the detection by qPCR was not 100% concordant with and *pfpm2/3* breakpoint assay for B5 and B5*-*r*exo*-E415G-B8 lines as qPCR assay showed the *pfpm3* multiple copy for Cambodia isolates, which was different from the previous report by Ansbro et al. [45]. It is not lost on the authors that different primers were used to identify the copy number of *pfpm3* and further investigation could be done. Therefore, it is important to also apply the breakpoint assay to isolates with copy number values < 1.52 for *pfpm2*, to capture all isolates with *pfpm2/3* amplification.

IC_90_ values, PPQ survival rates, and bimodal dose-response curves were used for assessing PPQ resistance in vitro [16, 17, 48]. All three assays confirmed that the engineered parasites harbouring the E415G-PfEXO mutation (B5-r*exo*-E415G-B8) did not show a PPQ resistant phenotype. On the contrary, parasites with the combination of PfEXO-E415G mutation with either novel PfCRT mutations or *pfpm2* multiple copies demonstrated reduced IC_90_ susceptibility, high PPQ survival rates, and a second peak of bimodal curve (Fig. 3). If the PfEXO-E415G mutation does not alter the PPQ susceptibility, this finding also recapitulates the work of Silva et al. [29] that parasites with the *pfpm2* multiple copy alone (i.e. ASAP-168) show reduced PPQ susceptibility and involved in PPQ resistance. Nonetheless, when the novel PfCRT mutation was added on top of the *pfpm2* multiple copies, the level of PPQ resistance increased tremendously as judged by the IC_50_ and IC_90_ values as well as AUC between 0.01 and 100 µM of PPQ in the bimodal dose-response curve. Bopp et al. [16] showed that when exposed up to 10 µM PPQ for 12 h, PPQ resistance parasites could survive and complete their lifecycle. It was also previously evident that the introduction of novel PfCRT mutations resulted in a fitness cost for the mutations [19, 20]. Similar trends were observed in this study; parasites holding either novel PfCRT mutations or *pfpm2* multiple copies grew much slower than those without mutations. It is likely that introducing PfEXO-E415G mutations may account for better fitness compared to parasites with the same genetic background. Even though the scope of this work could not address the function of PfEXO, it is evidently showed that the E415G mutation in PfEXO does not alter PPQ susceptivity, but rather affects parasite fitness.

## Conclusions

In summary, this study suggests that the E415G mutation in PfEXO could still be an important marker if used in conjunction with other markers. The insertion of the PfEXO-E415G mutation did not lead to an increased PPQ-IC_90_ or improve %PPQ survival, suggesting that this mutation is not associated with PPQ resistance. Additionally, this specific mutation resulted in parasites that grew better than those with the same background, highlighting the importance of genetic mutations toward parasite fitness and resistance.

## Supplementary Information

**Additional file 1: Table S1.** Oligonucleotideprimer sequences used in this study. **Figure S1.** DNA sequence alignment of* exo* gene from *P. falciparum *3D7,B5 and B5-r*exo*-E415G-B8 parasites. **Figure S2.** Gene and amino acid composition of PfEXO. **Figure S3.** Schematic of *P. falciparum plasmepsin* 2/3 (*pfpm2*/3)gene duplication.

## Data Availability

All data generated or analysed during this study are included in this published article and its Additional files.
