# Supplementary material for: A single point mutation in the Plasmodium falciparum 3′–5′ exonuclease does not alter piperaquine susceptibility
Source: Malar J. 2022 Apr 22;21:130. doi: 10.1186/s12936-022-04148-z (PMC9034581; doi:10.1186/s12936-022-04148-z)
Supplement: Supplementary file 1 — Additional file 1: Table S1. Oligonucleotideprimer sequences used in this study. Figure S1. DNA sequence alignment of exo gene from P. falciparum 3D7,B5 and B5-rexo-E415G-B8 parasites. Figure S2. Gene and amino acid composition of PfEXO. Figure S3. Schematic of P. falciparum plasmepsin 2/3 (pfpm2/3)gene duplication. [file 12936_2022_4148_MOESM1_ESM.docx]

**Additional file 1**

**A single point mutation in the *Plasmodium falciparum* 3´-5´exonuclease does not alter piperaquine susceptibility**

Nonlawat Boonyalai^1*^, Kirakarn Kirativanich^1^, Chatchadaporn Thamnurak^1^, Chantida Praditpol^1^, Brian A. Vesely^1^, Mariusz Wojnarski^1^, John S. Griesenbeck^1^, Norman C. Waters^1^.

^1^Department of Bacterial and Parasitic Diseases, Armed Forces Research Institute of Medical Sciences, Bangkok, Thailand.

Short running title: The E145G mutation in the exonuclease gene does not confer piperaquine resistance in *Plasmodium falciparum*

*Correspondence: Nonlawat Boonyalai, nonlwatb.fsn@afrims.org

**Table S1** Oligonucleotide primer sequences used in this study

| **Primer name** | **Sequence (5′ - 3′)** |
| --- | --- |
| Q5SDM_G415E_F | AAGAGGTGAACAACCACTGGGAG |
| Q5SDM_G415E_R | CCTTGATGGTGATCACCATATTGTTAAAATC |
| Bsd_NcoI_F | GGATCCATGGCCAAGCCTTTGTCTCAAG |
| Bsd_SacII_R | AATTCCGCGGCCGCGGTTAGCCCTCCCACACATAAC |
| sgE415G-1F | ATTGTATGGTTATAACGATAAAAG |
| sgE415G-1R | AAACCTTTTATCGTTATAACCATA |
| ExonI_K283_F **(Primer 1)** | AAGCAGTTGAAAAATACACTCTTTTTATTATTAAACGATATTGAGTG |
| Screen_WT_Rev_V616 **(Primer 2)** | AATCTATTATATATATTTTATTATTTGTTGAAATAGATAGAACACTA |
| Recodon_F **(Primer 4)** | ATGGTGATCACCATCAAGGGCG |
| Recodon_R **(Primer 3)** | TTCCAGCAGCCACTTGAAGAATGT |
| Screen_3 ′UTR_R **(Primer 5)** | CGAGGGGATAAGGTTTATTTTGTAGGGAAAAG |
| Pfk13_outer_F | CGGAGTGACCAAATCTGGGA |
| Pfk13_outer_R | GGGAATCTGGTGGTAACAGC |
| Pfk13_nested_F | GCCAAGCTGCCATTCATTTG |
| Pfk13_nested_R | GCCTTGTTGAAAGAAGCAGA |
| Pfexo_E415G_F | GCACCTCCTATCATCAGATGATACC |
| Pfexo_E415G_R | CAAGAAAAGAGGAAGGAACACCTTC |
| Pfcrt_93_145_F | TGCTAAAAGAACTTTAAACAAAATTGG |
| Pfcrt_93_145_R | CAAGAACTACTGGAAATATCCAATC |
| Pfcrt_218_F | TCTCGGAGCAGTTATTATTGTTG |
| Pfcrt_218_R | ATTTCCCTTGTCATGTTTGAA |
| Pfcrt_343_353_F | CGCATTGTTTTCCTTCTTTAAC |
| Pfcrt_343_353_R | CGGCTAAGAATTTAAAGTAATAAGCAA |
| Pfpm1_fw | CAATGGTTTCGAACCAGCTT |
| Pfpm1_rv | GGTAAAAACGGCTTGTTCGAT |
| Pfpm2_fw | TGGTGATGCAGAAGTTGGAG |
| Pfpm2_rv | TGGGACCCATAAATTAGCAGA |
| Pfpm3_fw | CACCTTCATGAAAAATGAAGAATC |
| Pfpm3_rv | AAGAAAAACCTCCTGCCAAAA |
| PfB-tub_fw | TGATGTGCGCAAGTGATCC |
| PfB-tub_rv | TCCTTTGTGGACATTCTTCCTC |
| AF_for | CCACGATTTATATTGGCAAGTTGATTTAG |
| AR_rev | CATTTCTACTAAAATTAGCTTTAGCATCATTCACG |
| BF_for | CGTAGAATCTGCAAGTGTTTTCAAAG |
| BR_rev | AATGTTATAAATGCAATATAATCAAACGACATCAC |

(Coloured arrows in the left-hand column correspond to primer annotation in Fig 1)

PF3D7_Exo_WT ATGACATCACATATTAGTTACAATAAAATAAGAGAAAAAAGGAACATAAGAAGGGTACTG 60

PFB5_Exo_WT ATGACATCACATATTAGTTACAATAAAATAAGAGAAAAAAGGAACATAAGAAGGGTACTG 60

PFB8_Exo_E415G ATGACATCACATATTAGTTACAATAAAATAAGAGAAAAAAGGAACATAAGAAGGGTACTG 60

************************************************************

PF3D7_Exo_WT AGCGTTTATAATTTTTGTTCATTAAATAGATTCTCATGGTACAGACAAGATGTGAGATTA 120

PFB5_Exo_WT AGCGTTTATAATTTTTGTTCATTAAATAGATTCTCATGGTACAGACAAGATGTGAGATTA 120

PFB8_Exo_E415G AGCGTTTATAATTTTTGTTCATTAAATAGATTCTCATGGTACAGACAAGATGTGAGATTA 120

************************************************************

PF3D7_Exo_WT GATAATGTGATATATAGAAAGAATTATGAATATATTTATTATAATTGTATTATAAGAAAA 180

PFB5_Exo_WT GATAATGTGATATATAGAAAGAATTATGAATATATTTATTATAATTGTATTATAAGAAAA 180

PFB8_Exo_E415G GATAATGTGATATATAGAAAGAATTATGAATATATTTATTATAATTGTATTATAAGAAAA 180

************************************************************

PF3D7_Exo_WT TGTAGTAGAAATTATATATCTACACGTAATAATATATATATTAAAAACAAAATATATGAT 240

PFB5_Exo_WT TGTAGTAGAAATTATATATCTACACGTAATAATATATATATTAAAAACAAAATATATGAT 240

PFB8_Exo_E415G TGTAGTAGAAATTATATATCTACACGTAATAATATATATATTAAAAACAAAATATATGAT 240

************************************************************

PF3D7_Exo_WT ATAATACCATATTTATGTAAAGGTAAAGATGTAAAAAATATAACAAGTAATATAATATTT 300

PFB5_Exo_WT ATAATACCATATTTATGTAAAGGTAAAGATGTAAAAAATATAACAAGTAATATAATATTT 300

PFB8_Exo_E415G ATAATACCATATTTATGTAAAGGTAAAGATGTAAAAAATATAACAAGTAATATAATATTT 300

************************************************************

PF3D7_Exo_WT TATATTTTACAACATTTAAGTACAAAAAATGTTGTTAATAGTAATGAATATAAAGATAAT 360

PFB5_Exo_WT TATATTTTACAACATTTAAGTACAAAAAATGTTGTTAATAGTAATGAATATAAAGATAAT 360

PFB8_Exo_E415G TATATTTTACAACATTTAAGTACAAAAAATGTTGTTAATAGTAATGAATATAAAGATAAT 360

************************************************************

PF3D7_Exo_WT ATAAAAAAAATATATTTTAATTTATTGAAATGTTATCATAAAATTTTTGAATATAATAAT 420

PFB5_Exo_WT ATAAAAAAAATATATTTTAATTTATTGAAATGTTATCATAAAATTTTTGAATATAATAAT 420

PFB8_Exo_E415G ATAAAAAAAATATATTTTAATTTATTGAAATGTTATCATAAAATTTTTGAATATAATAAT 420

************************************************************

PF3D7_Exo_WT GAATATGGAGAATATATATTTAGCCTGTTTAATGATGATATAATTATATCTGTTAGTCCG 480

PFB5_Exo_WT GAATATGGAGAATATATATTTAGCCTGTTTAATGATGATATAATTATATCTGTTAGTCCG 480

PFB8_Exo_E415G GAATATGGAGAATATATATTTAGCCTGTTTAATGATGATATAATTATATCTGTTAGTCCG 480

************************************************************

PF3D7_Exo_WT TCTTTAAGAAAGAAACAAAAAAATATTATACAAGATATATTATTGAATAGCTTACATTTT 540

PFB5_Exo_WT TCTTTAAGAAAGAAACAAAAAAATATTATACAAGATATATTATTGAATAGCTTACATTTT 540

PFB8_Exo_E415G TCTTTAAGAAAGAAACAAAAAAATATTATACAAGATATATTATTGAATAGCTTACATTTT 540

************************************************************

PF3D7_Exo_WT TTTTTCCAGAATAATATAACATATAAAAATAAATTAAATATAAATTTGATGTGTGAAATT 600

PFB5_Exo_WT TTTTTCCAGAATAATATAACATATAAAAATAAATTAAATATAAATTTGATGTGTGAAATT 600

PFB8_Exo_E415G TTTTTCCAGAATAATATAACATATAAAAATAAATTAAATATAAATTTGATGTGTGAAATT 600

************************************************************

PF3D7_Exo_WT ATTAATTATCCAAAGTTTTTGTATGTATTACATTCTATTAATTATGATATGAATATATTA 660

PFB5_Exo_WT ATTAATTATCCAAAGTTTTTGTATGTATTACATTCTATTAATTATGATATGAATATATTA 660

PFB8_Exo_E415G ATTAATTATCCAAAGTTTTTGTATGTATTACATTCTATTAATTATGATATGAATATATTA 660

************************************************************

PF3D7_Exo_WT AAACAAAATATAAGTGTACAAAATGTAGATTATTTATTTTCTCAGTATATTAATAAAACA 720

PFB5_Exo_WT AAACAAAATATAAGTGTACAAAATGTAGATTATTTATTTTCTCAGTATATTAATAAAACA 720

PFB8_Exo_E415G AAACAAAATATAAGTGTACAAAATGTAGATTATTTATTTTCTCAGTATATTAATAAAACA 720

************************************************************

PF3D7_Exo_WT AATATATATATTACTACAGCAATTCAATTCGCTTCATTTTTTAAAAATGTCAACATGAAT 780

PFB5_Exo_WT AATATATATATTACTACAGCAATTCAATTCGCTTCATTTTTTAAAAATGTCAACATGAAT 780

PFB8_Exo_E415G AATATATATATTACTACAGCAATTCAATTCGCTTCATTTTTTAAAAATGTCAACATGAAT 780

************************************************************

PF3D7_Exo_WT ATTTTCACACCTTTTAAGAAACACGGTACATTTAATTATTTCTTATTATTAAAACGTATA 840

PFB5_Exo_WT ATTTTCACACCTTTTAAGAAACACGGTACATTTAATTATTTCTTATTATTAAAACGTATA 840

PFB8_Exo_E415G ATTTTCACACCTTTTAAGAAACACGGTACATTTAATTATTTCTTATTATTAAAACGTATA 840

************************************************************

PF3D7_Exo_WT GTAAATAAGCAGTTGAAAAATACACTCTTTTTATTATTAAACGATATTGAGTGTCATCGG 900

PFB5_Exo_WT GTAAATAAGCAGTTGAAAAATACACTCTTTTTATTATTAAACGATATTGAGTGTCATCGG 900

PFB8_Exo_E415G GTAAATAAGCAGTTGAAAAATACACTCTTTTTATTATTAAACGATATTGAGTGTCATCGG 900

************************************************************

PF3D7_Exo_WT TTAAGGCAGGAAATGCTTTTGCACCTCCTATCATCAGATGATACCACAGgtataaaaaaa 960

PFB5_Exo_WT TTAAGGCAGGAAATGCTTTTGCACCTCCTATCATCAGATGATACCACAGgtataaaaaaa 960

PFB8_Exo_E415G TTAAGGCAGGAAATGCTTTTGCACCTCCTATCATCAGATGATACCACAGgtataaaaaaa 960

************************************************************

PF3D7_Exo_WT aaaaaaatatatatatatatatatatatatttatatatgtatatatgtatatatgtatat 1020

PFB5_Exo_WT aaaaaaatatatatatatatatatatatatttatatatgtatatatgtatatatgtatat 1020

PFB8_Exo_E415G aaaaaaatatatatatatatatatatatatttatatatgtatatatgtatatatgtatat 1020

************************************************************

PF3D7_Exo_WT atttattttttatttattatttttttttttttttttttttttccttctgacccctttata 1080

PFB5_Exo_WT atttattttttatttattatttttttttttttttttttttttccttctgacccctttata 1080

PFB8_Exo_E415G atttattttttatttattatttttttttttttttttttttttccttctgacccctttata 1080

************************************************************

PF3D7_Exo_WT gGAATGTGCTTTAACGAATGGAGTCATTTAGCAGCAAAGAAATATTTACTTATGAACAAG 1140

PFB5_Exo_WT gGAATGTGCTTTAACGAATGGAGTCATTTAGCAGCAAAGAAATATTTACTTATGAACAAG 1140

PFB8_Exo_E415G gGAATGTGCTTTAACGAATGGAGTCATTTAGCAGCAAAGAAATATTTACTTATGAACAAG 1140

************************************************************

PF3D7_Exo_WT TATGAAAATGATCTTAAGGAAAATATAAGAGATCAAGAAATAAATGTTAACATTTTGAAA 1200

PFB5_Exo_WT TATGAAAATGATCTTAAGGAAAATATAAGAGATCAAGAAATAAATGTTAACATTTTGAAA 1200

PFB8_Exo_E415G TATGAAAATGATCTTAAGGAAAATATAAGAGATCAAGAAATAAATGTTAACATTTTGAAA 1200

************************************************************

PF3D7_Exo_WT AGATCAATTGATTATGTTAAGGATTACGATGACGATAACGATAAAGTTAACAATAACGAT 1260

PFB5_Exo_WT AGATCAATTGATTATGTTAAGGATTACGATGACGATAACGATAAAGTTAACAATAACGAT 1260

PFB8_Exo_E415G AGATCAATTGATTATGTTAAGGATTACGATGACGATAACGATAAAGTTAACAATAACGAT 1260

************************************************************

PF3D7_Exo_WT AACGATAATGATAACGATTTATATATGGAATATTTTAATTTACCTGAAGACGTTAAAAAT 1320

PFB5_Exo_WT AACGATAATGATAACGATTTATATATGGAATATTTTAATTTACCTGAAGACGTTAAAAAT 1320

PFB8_Exo_E415G AACGATAATGATAACGATTTATATATGGAATATTTTAATTTACCTGAAGACGTTAAAAAT 1320

************************************************************

PF3D7_Exo_WT GTAAAGTACATAAAATGTGTTGATGATTTTAACAATATGGTTATAACGATAAAA**GAG**GAA 1380

PFB5_Exo_WT GTAAAGTACATAAAATGTGTTGATGATTTTAACAATATGGTTATAACGATAAAA**GAG**GAA 1380

PFB8_Exo_E415G GTAAAGTACATAAAATGTGTTGATGATTTTAACAATATGGTGATCACCATCAAG**GGC**GAG 1380

***************************************** ** ** ** ** * **

PF3D7_Exo_WT GTAAACAATCATTGGGAAAATAATATATACAATAAAAAGGATATGGTTAATTATACAAAT 1440

PFB5_Exo_WT GTAAACAATCATTGGGAAAATAATATATACAATAAAAAGGATATGGTTAATTATACAAAT 1440

PFB8_Exo_E415G GTGAACAACCACTGGGAGAACAACATCTACAACAAGAAGGACATGGTCAACTACACCAAT 1440

** ***** ** ***** ** ** ** ***** ** ***** ***** ** ** ** ***

PF3D7_Exo_WT GAAAATTATAATGATAATATATTAACATATGAATATATTAATGAAACACTTAGAAAAGAA 1500

PFB5_Exo_WT GAAAATTATAATGATAATATATTAACATATGAATATATTAATGAAACACTTAGAAAAGAA 1500

PFB8_Exo_E415G GAGAACTACAACGACAACATCCTGACCTACGAGTACATCAACGAGACACTGAGAAAGGAA 1500

** ** ** ** ** ** ** * ** ** ** ** ** ** ** ***** ***** ***

PF3D7_Exo_WT AAAAAAAGATATTATATAGGTATAGATATCGAATGGGATAGTTATAAAAAAAAAAAAAAT 1560

PFB5_Exo_WT AAAAAAAGATATTATATAGGTATAGATATCGAATGGGATAGTTATAAAAAAAAAAAAAAT 1560

PFB8_Exo_E415G AAGAAGCGGTACTACATCGGCATCGACATCGAGTGGGACAGCTACAAGAAAAAGAAAAAC 1560

** ** * ** ** ** ** ** ** ***** ***** ** ** ** ***** *****

PF3D7_Exo_WT ACTGTTAGTGTTCTATCTATTTCAACAAATAATAAAATATATATAATAGATTTATATTAT 1620

PFB5_Exo_WT ACTGTTAGTGTTCTATCTATTTCAACAAATAATAAAATATATATAATAGATTTATATTAT 1620

PFB8_Exo_E415G ACCGTGTCCGTGCTGAGCATCAGCACCAACAACAAGATCTACATCATCGATCTGTACTAC 1620

** ** ** ** ** ** ** ** ** ** ** ** ** *** * ** **

PF3D7_Exo_WT ATTGATTATAATTATAAATTTATGATATATACGTTTTTTAAATGGTTATTAGAAAATCCG 1680

PFB5_Exo_WT ATTGATTATAATTATAAATTTATGATATATACGTTTTTTAAATGGTTATTAGAAAATCCG 1680

PFB8_Exo_E415G ATCGACTACAATTACAAGTTCATGATCTATACATTCTTCAAGTGGCTGCTGGAAAATCCG 1680

** ** ** ***** ** ** ***** ***** ** ** ** *** * * *********

PF3D7_Exo_WT TTTATATATAAATTGTTTTTTAATTTCCCTTCGGATATTAAAATAATGTCTTCATATTTT 1740

PFB5_Exo_WT TTTATATATAAATTGTTTTTTAATTTCCCTTCGGATATTAAAATAATGTCTTCATATTTT 1740

PFB8_Exo_E415G TTTATATATAAATTGTTTTTTAATTTCCCTTCGGATATTAAAATAATGTCTTCATATTTT 1740

************************************************************

PF3D7_Exo_WT CAAAACATATCACATATAAATATATATAACAATATTATAGATTTAAATAATAATATATAT 1800

PFB5_Exo_WT CAAAACATATCACATATAAATATATATAACAATATTATAGATTTAAATAATAATATATAT 1800

PFB8_Exo_E415G CAAAACATATCACATATAAATATATATAACAATATTATAGATTTAAATAATAATATATAT 1800

************************************************************

PF3D7_Exo_WT ATATATACAAGAAAAGAGGAAGGAACACCTTCTTATAAGAATTATAATATTTTATATTTT 1860

PFB5_Exo_WT ATATATACAAGAAAAGAGGAAGGAACACCTTCTTATAAGAATTATAATATTTTATATTTT 1860

PFB8_Exo_E415G ATATATACAAGAAAAGAGGAAGGAACACCTTCTTATAAGAATTATAATATTTTATATTTT 1860

************************************************************

PF3D7_Exo_WT GAAACATTGAATAGAGATATGATTCAATCAAATGATGTACATTTATTCAAAGAATTAGTA 1920

PFB5_Exo_WT GAAACATTGAATAGAGATATGATTCAATCAAATGATGTACATTTATTCAAAGAATTAGTA 1920

PFB8_Exo_E415G GAAACATTGAATAGAGATATGATTCAATCAAATGATGTACATTTATTCAAAGAATTAGTA 1920

************************************************************

PF3D7_Exo_WT CATTCGACACCTTATAATTTTAATAAGAATTTAATGAATAAAATAAAAAAA**AAA**AATAAT 1980

PFB5_Exo_WT CATTCGACACCTTATAATTTTAATAAGAATTTAATGAATAAAATAAAAAAA**AAT**AATAAT 1980

PFB8_Exo_E415G CATTCGACACCTTATAATTTTAATAAGAATTTAATGAATAAAATAAAAAAA**AAT**AATAAT 1980

***************************************************** ******

PF3D7_Exo_WT AATATTAATATACCAAACAAACAAATGTTCAAATTATATGTTAAAAGTTTAAATGACTTA 2040

PFB5_Exo_WT AATATTAATATACCAAACAAACAAATGTTCAAATTATATGTTAAAAGTTTAAATGACTTA 2040

PFB8_Exo_E415G AATATTAATATACCAAACAAACAAATGTTCAAATTATATGTTAAAAGTTTAAATGACTTA 2040

************************************************************

PF3D7_Exo_WT TGTATAAAGATATTAAATAAAAAGTTAAATAAAAAATTTCAATTAGCTAACTGGAATATA 2100

PFB5_Exo_WT TGTATAAAGATATTAAATAAAAAGTTAAATAAAAAATTTCAATTAGCTAACTGGAATATA 2100

PFB8_Exo_E415G TGTATAAAGATATTAAATAAAAAGTTAAATAAAAAATTTCAATTAGCTAACTGGAATATA 2100

************************************************************

PF3D7_Exo_WT AGACCACTTAACCAAGAACAAATAATATATGCATGTATTGATTCATATGTTTTAATAAAG 2160

PFB5_Exo_WT AGACCACTTAACCAAGAACAAATAATATATGCATGTATTGATTCATATGTTTTAATAAAG 2160

PFB8_Exo_E415G AGACCACTTAACCAAGAACAAATAATATATGCATGTATTGATTCATATGTTTTAATAAAG 2160

************************************************************

PF3D7_Exo_WT ATAGAAGAAATGTTGATAGAAAAAGGTTATATGTCTACATGTGATTCTAACAACAATCAA 2220

PFB5_Exo_WT ATAGAAGAAATGTTGATAGAAAAAGGTTATATGTCTACATGTGATTCTAACAACAATCAA 2220

PFB8_Exo_E415G ATAGAAGAAATGTTGATAGAAAAAGGTTATATGTCTACATGTGATTCTAACAACAATCAA 2220

************************************************************

PF3D7_Exo_WT ATGATGAATTTATTTCTTCAAAAATATAAGTTCAAAGATAGTACATGGGAATGA 2274

PFB5_Exo_WT ATGATGAATTTATTTCTTCAAAAATATAAGTTCAAAGATAGTACATGGGAATGA 2274

PFB8_Exo_E415G ATGATGAATTTATTTCTTCAAAAATATAAGTTCAAAGATAGTACATGGGAATGA 2274

******************************************************

**Fig.S1.** **DNA sequence alignment of *exo* gene from *P. falciparum* 3D7, B5 and B5-r*exo*-E415G-B8 parasites.** The *exo* gene contains 2 exons and one intron. Exon 1 starts from nucleotides 1 to 949, while exon 2 starts from nucleotides 1018 to 2274. The intron is shown in lowercase letters. Grey highlight indicates the recodonized region in B8 line with the amino acid residue 415 change from Glu (codon GAG) to Gly (codon GGC). One single nucleotide mutation at position 1974 from A to T leads to non-synonymous mutation, changing from amino acid Lys to Asn. Guide RNA sequence is shown in yellow highlight while the Protospacer Adjacent Motif (PAM) site is shown in magenta highlight.


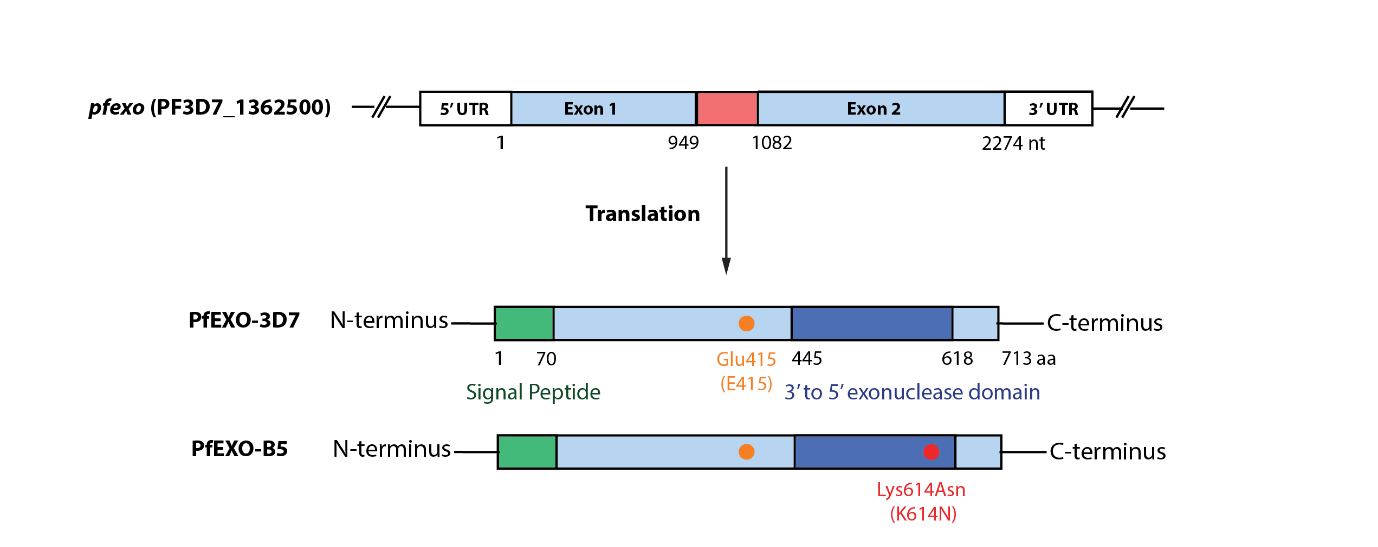


**Fig. S2.** **Gene and amino acid composition of PfEXO**. Schematic diagram of *pfexo* gene and domain structure of PfEXO from 3D7 and B5 lines. *pfexo* gene contains 2 exons (light blue boxes) and 1 intron (red box). PfEXO contains N-terminal signal peptide (green box) and 3′ to 5′ exonuclease domain (navy blue box). Single amino acid mutation from Lys to Asn at residue 614 found in PfEXO from the B5 line is indicated in red circles. The amino acid Glu at residue 415, focused on in this study, is indicated in orange circles.


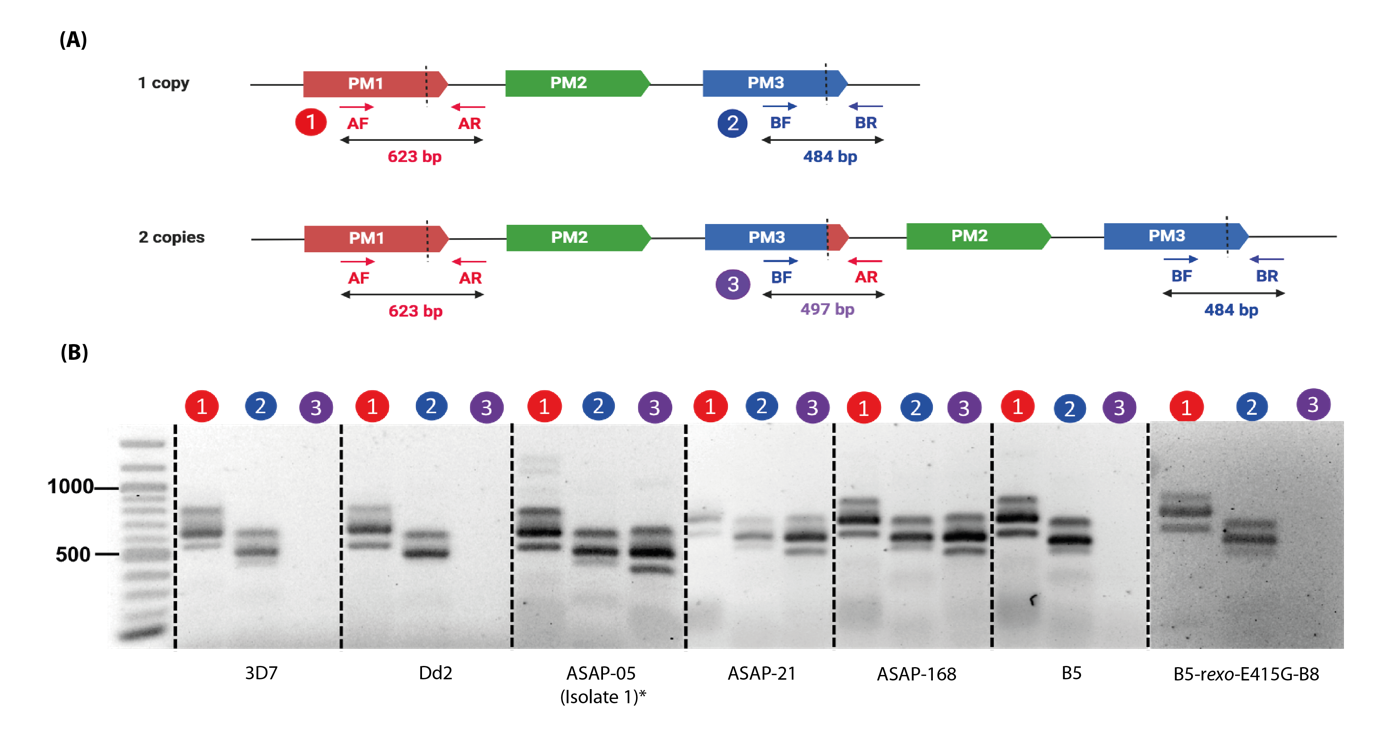


**Fig S3**. Schematic of *P. falciparum plasmepsin* 2/3 *(pfpm2*/3) gene duplication. (A) Gene model showing the *pfpm* 2/3 breakpoint (dashed lines). Primer positions are labelled in the single copy (top) and multiple copy (bottom) isolates. (B) Amplification primer pairs 1 and 2 are to confirm that regions surrounding the *pfpm* 2/3 amplification are present in all samples, while primer pair 3 is to detect the *pfpm* 2/3 amplification and no PCR products are observed in samples with a single copy of *pfpm* 2/3 amplification. It is noted that ASAP-05 was name isolate 1 in the previous publication^1^.

**Reference**

1 Boonyalai, N. *et al.* Piperaquine resistant Cambodian Plasmodium falciparum clinical isolates: in vitro genotypic and phenotypic characterization. *Malar J* **19**, 269, doi:10.1186/s12936-020-03339-w (2020).
